# Nitrite of Amyl in Uterine Hemorrhage

**Published:** 1880-02

**Authors:** 


Nitrite of Amyl in Uterine Hemorrhage.—Mr. Kerr adds his testimony to the usefulness of this remedy (*British Medical Journal*, vol. ii., 1879, p. 691). He considers the rationale of its effect to be rapid dilatation of the cervical vessels. Attending a rather severe case of post-partum hémorrhage, Dr. Kerr administered five minims of the nitrite by means of an inhaler, with immediate and happy results. The hemorrhage ceased at once and permanently, and the patient was restored from a state of collapse.—*Philadelphia Medical Times.*

